# Clinical Society of Maryland

**Published:** 1892-10

**Authors:** W. T. Watson

**Affiliations:** Sec’y; 1519 N. Broadway, Baltimore, Md.


### CLINICAL SOCIETY OF MARYLAND.

BALTIMORE, MD., May 20, 1892.

The 267th regular meeting was called to order by the President, Dr. Robt. W. Johnson.

DR. H. O. REIK, 1525 N. Caroline St., Baltimore, was elected to membership.

DR. SAMUEL THESBALD related "A Case in which the Electro-Magnet was Employed Successfully for the Removal of a Fragment of Steel from the Vitreous Chamber of the Eye."

A lad, of 12 years of age, while using a hammer, struck a small piece of steel, which penetrated the eye and lodged in the vitreous chamber. The case was first seen six days after the accident. The fragment penetrated the upper margin of the cornea, and just in line with this was a hole through the iris as large as a small pin's-head. The eye was markedly injected with evidences of, perhaps, commencing iritis. In vitreous humor, diffused opacity and numerous floating opacities. There was a punctate opacity on the anterior surface of the lens where it had been touched by the foreign body. Details of fundus could not be seen. The foreign body was not visible. Vision, 16-125ths. Operation five days after the patient was first seen or eleven days after the accident. The injection increased and iritis had begun. Incision about 4 m. m. in length through the sclerotic between the external and inferior rectus muscles. A Hirshberg's electro-magnet was employed. A single cell of the battery was used; this enabled the magnet to lift up a tack hammer. The point of the magnet was introduced well into the vitreous humor three or four times without success, but finally it brought out the little particle of steel, the size of a pin's-head. The conjunctival wound was stitched, and an opium and boracic acid lotion with compress was used. Atropia kept the pupil dilated. Boy suffered very little. Seventeen days after the operation he left the hospital, at which time the injection was very much less, the vitreous had cleared up very materially and vision was 16-45ths. At the present time, forty-four days after operation, the fundus of the eye can be seen with perfect ease. There are one or two floating opacities in the vitreous humor Vision 16-30ths.

DR. ROBERT RANDOLPH: This case is one of a very large class, forming the larger number of cases which come to us for enucleation, and the larger number which end in sympathetic opththalmia. We have there a better method of dealing with such cases. When we have a reasonable idea of the location of the foreign body, and under strict antiseptic precautions, the operation is indicated, and there are a sufficient number of cases on record to justify us in looking for a happy issue.

DR. KATE CAMPBELL HURD read a paper on "Treatment of Spinal Curvature by the Zander Method."

DR. J. H. BRANHAM reported a case in which "A Sea-Tangle Tent was Forced into Douglas' Cul-de-sac in an Attempt to Produce Abortion."

On February 27, 6 P. M., saw in consultation a young married woman of 24, mother of three children. She had been about two months pregnant and had attempted to produce abortion on herself with a sea-tangle tent three days before I saw her. After leaving it for twenty-four hours, she tried to remove it, but simply pulled out the string. Next morning her physician was summoned, but failed to find the tent, although the uterus was partly dilated, and from it issued a bad smelling discharge. When I saw her, her temperature was 103, pulse 120, abdomen very much swollen and exceedingly tender. The finger could be introduced into the uterine cavity, but no tent was found. An opening in the wall of the cervix was discovered, and through this the tent was felt in Douglas' cul-de-sac. It was removed through this opening, and was found to be about the size of one's little finger. An opening was made into the cul-de-sac, and a drainage tube put in. The uterus and vagina were washed out with 1 4000 bichloride. There was a temporary improvement, but she finally died, thirty-six hours after I first saw her.

The woman maintained to the last that she introduced the tent herself, and this is probably true, considering the direction in which it was forced.

W. T. WATSON, M. D , Sec'y.

1519 N. Broadway, Baltimore, Md.

